# Physical Fitness Profiling of National Category Table Tennis Players: Implication for Health and Performance

**DOI:** 10.3390/ijerph18179362

**Published:** 2021-09-04

**Authors:** Jon Mikel Picabea, Jesús Cámara, Javier Yanci

**Affiliations:** 1 Physical Education and Sport Department, Faculty of Education and Sport, University of the Basque Country UPV/EHU, 01007 Vitoria-Gasteiz, Spain; jmpicabea002@ehu.eus; 2 Society, Sports and Physical Exercise Research Group (GIKAFIT), Physical Education and Sport Department, Faculty of Physical Activity and Sports Science, University of the Basque Country, UPV/EHU, 01007 Vitoria-Gasteiz, Spain; jesus.camara@ehu.eus

**Keywords:** sprint, strength, power, flexibility, indoor, racket sports

## Abstract

The aims of this study were to: (1) analyze table tennis players’ physical profiles considering and comparing players age categories (i.e., under U12, U14, U16, U20, Senior and Older); and (2) to quantify the correlations among the variables measured by each test. Seventy-one table tennis players (61 men and 10 women, 19.7 ± 11.23 years, 1.65 ± 0.13 m, 59.71 ± 17.72 kg and 21.60 ± 4.22 kg/m^2^) divided into six age groups, performed a sprint test, forearm isometric strength test, countermovement vertical test, countermovement horizontal test, change of direction ability (CODA) test and flexibility test. U14 players performed better than U12 in all tests (ES = −0.70 to 1.98, moderate to large) except in Sit and Reach (SAR) test (ES = 0.19, trivial). The U16 group also obtained better results than U14 in all tests (ES = 0.77 to −2.31, moderate to large) except for the SAR test (ES = 0.19, trivial). The U20 group performed better than U16 in all the tests (ES = 0.73 to −1.53, moderate to large) except for the 5 m sprint test (ES = −0.02, trivial), 10 m sprint test (ES = −0.51, moderate) and SAR (ES = 0.11, trivial). Differences between Senior and U20 were only found in the arm swing counter movement jump (CMJAS) (ES = −0.82, large) and modified agility test (MAT) (ES = 1.19, large), with the U20 group being better in both variables. The senior group performed better in the MAT test than the older group (ES = 0.94, large). The relation found between forearm isometric strength, vertical jump, horizontal jump, sprint and CODA ability (r = −0.53; ±0.14, 0/0/100, most likely to r = 0.83; ±0.06, 100/0/0, most likely) indicates that these capacities are related in table tennis players. Nevertheless, the lack of association between the sit and reach test with the other capacities may indicate that flexibility is an independent capacity.

## 1. Introduction

Despite the fact that table tennis is not a majority sport in many countries, about 300 million individuals participate in table tennis [1], of whom at least 40 million are competitive players [2]. As in other sport disciplines [3,4], it is important to know the health status of the athletes, especially in amateur category players who usually cannot count on being monitored by qualified personnel in training sessions and competitions. If health status can be analyzed from different dimensions (physical-physiological, psychological, emotional status) [5], some health benefits have been found in table tennis [6], and an adequate physical condition can be related to better health levels [7] in different age populations. Specifically, the benefits of playing table tennis are related with hand–eye coordination, balance, coordination, brain stimulation and development of cognitive functions, development of body composition, and improving fat distribution [1]. Furthermore, as previous studies mentioned before, children who regularly play table tennis have greater bone development and superior physical fitness (strength, range of movement, and cardiovascular fitness) compared to those who are physically active, while benefits in muscle strength and neuromotor skills are shown to be maintained in older people [8]. Besides, this sport has been recommended as a tool for increasing physical activity [6].

Table tennis players are required to hit the ball over 30 times per minute during rallies no longer than 4 s, with resting times shorter than 15 s [9], with the ball travelling at high speed (>50 km·h^−1^), forcing players to respond in milliseconds [8]. Consequently, many table tennis experts have pointed out that motor skills and physical fitness are important traits in table tennis [10]. Concretely, high levels of speed, agility, coordination, reaction time, strength, and flexibility are essential to perform the different techniques correctly [8,9,10,11]. In order to study the physical capacities required in different sports, many tests have been used to assess upper and lower body strength, change of direction ability (CODA), flexibility and acceleration, among others [8,11,12,13,14,15]. Specifically, previous studies have analyzed the differences in these capacities in different age groups of table tennis players [8,16,17]. In particular, Pradas et al. [8] found that 10–11-years-old table tennis players performed better in upper limb strength, range of motion of the lower back and cardiovascular fitness than physical active but not engaged in a regular physical activity. Similarly, Faber et al. [16] found that 10-year-old table tennis players performed better in sprint, agility, speed while dribbling and throwing a ball test than 7-year-old table tennis players. In this same vein, Pradas de la Fuente et al. [17] observed a direct correlation between age and vertical jump capacity in the age range from 11 to 18 in table tennis players. Nevertheless, although in other sport disciplines older age range participants have been analyzed [18,19], only one study has been found that analyze differences in an older age range in table tennis players [8]. Concretely, this study determines the differences in anthropometric attributes of table tennis players according to sex, age, and ranking, concluding that higher lean mass in upper limbs was associated with higher ranking position and that ectomorphic profiles seem to be more related to performance, but no physical test was performed [8]. In order to ameliorate physical performance and minimize the risks of injuries, considering the biological changes occurring during maturation, it would be interesting to assess differences in physical tests among a broad range of age groups (i.e., from under 12 to seniors and older) [20].

Even though relationships among different physical capacities have been investigated in other sports [21,22], only two studies have reported this information in table tennis players [10,23]. Specifically, in table tennis, Faber et al. [23] found significant correlations between sprint, agility, and vertical jump tests. Nikolic et al. [10], studied the relationships between physical tests and specific table tennis tests. They found that some motor abilities (i.e., arm coordination, agility, explosive arm power, movement frequency speed, and repetitive leg power) had a positive influence in table tennis tests. However, whilst some studies observed significant correlations amongst physical variables in other sport disciplines, others did not find significant associations among the different tests [20,24,25,26]. It has been previously stated that age, sport discipline, and competitive level can determine the relationships among physical tests [10,26,27]; nevertheless, more studies are needed to analyze whether different physical capacities are inter-related or are independent from each other in table tennis players.

Therefore, the aims of the present study were to analyze the table tennis players’ physical profiles (i.e., handgrip isometric strength, vertical jump, horizontal jump, sprint, CODA, and flexibility) considering the age of the participants and to quantify the relationships among the physical tests. As a hypothesis, considering that previous studies have found significant age differences and significant correlations among sprint, agility, and strength tests, significant age differences in physical fitness profiling and significant correlations among these tests are expected in table tennis players.

## 2. Materials and Methods

### 2.1. Participants

Seventy-one table tennis players (61 men and 10 women, 19.7 ± 11.23 years, 1.65 ± 0.13 m, 59.71 ± 17.72 kg and 21.60 ± 4.22 kg/m^2^) divided into 6 age groups (Table 1) (under 12 (U12) (*n* = 11, 9 men and 2 women); under 14 (U14) (*n* = 13, 10 men and 3 women); under 16 (U16) (*n* = 11, 10 men and 1 women); under 20 (U20) (*n* = 14, 14 men and 0 women); between 20 and 30 years (Senior) (*n* = 12, 9 men and 3 women) and older than 30 years (Older) (*n* = 10, 9 men and 1 women)) participated in this study. Participants competed in any of the Basque Country (Spain) official categories. Inclusion criteria were as follows: (i) at least two years of competition experience at either the provincial or national level, (ii) training on a weekly basis (between 2 and 3 sessions per week, between 3 and 5 h per week). Exclusion criteria were: (i) players with severe pain or injury in the 6 months before the tests and (ii) taking medications. Written informed consent was obtained from the players and the club prior to the commencement of the study after a detailed written and oral explanation of the potential risks and benefits resulting from their participation. Written informed parental consent and player assent were obtained when players were under 18 years of age. Ethical approval was granted by the Ethics Committee for Research on Humans (CEISH, no. 2080310018-INB0059) of the University of the Basque Country (UPV/EHU) (in accordance with the Declaration of Helsinki 2013).

### 2.2. Procedures

Considering that table tennis requires high levels of speed, agility, strength, and flexibility [8,9,10,11], the table tennis players’ physical profiles were assessed on the basis of different capacities: isometric strength, vertical and horizontal jump, sprinting, CODA, and flexibility. Two test sessions were carried out during the midseason (i.e., March). On the first testing day, body mass and height were measured along with an assessment of a handgrip isometric strength test (HANDG), vertical countermovement jump with arm swing test (CMJAS), and horizontal countermovement jump with arm swing test (HCMJAS). On the second day, participants performed a 5 m and 10 m sprint test (S5M and S10M), a modified agility test (MAT) and a sit and reach test (SAR). These tests were chosen because they have been previously used in different racket sports [12,14,22,28,29]. Before each testing session a standardized warm-up consisting of 5 min of forehand and backhand topspin rallies was performed.

The tests were performed on an official table tennis court, in the usual training area, in the same time slot (18:00 and 20:00) during the mid-season, when the teams started the second half of the league. In the previous sessions, specific exercises were performed to familiarize the participants with the correct execution of the tests, giving explanations and concrete corrections.

### 2.3. Measures

Handgrip—Participants performed a maximum Handgrip test (HANDG), with a portable hydraulic hand dynamometer (5030J1, Jamar^®^, Sammons Preston, Inc., United Kingdom), with their dominant hand in extension and in the vertical axis [30]. The dominant hand was determined as the hand used to hold the table tennis racket [28]. The testing protocol consisted of three maximal isometric contractions for 5 s, with a rest period of 1 min between repetitions. Trials where the isometric contraction was less than 5 s or the arm was not fully extended, were repeated. Peak absolute strength (kg) was considered as the maximum isometric strength [28]. The highest value was considered for further analysis.

Vertical countermovement jumps with arm swing—Participants performed three consecutive vertical countermovement jumps with arm swing (CMJAS) interspersed with 45 s rest periods. Subjects had to reach the knee angle of 90º as quickly as possible and jump immediately afterwards [29]. Trials where the landing was not made with the legs fully extended were repeated. The flight time was measured with a platform (Optojump^®^, Microgate Engineering, Bolzano, Italy). The highest flight time value was considered for further analysis.

Horizontal countermovement jumps with arm swing—Participants performed three horizontal countermovement jumps with arm swing (HCMJAS) interspersed with 45 s rest periods [14,22,29]. Jump distance was calculated measuring the distance from the starting line to the landing point at heel contact [14] with a measuring tape. Trials where the heel moved from the landing point were repeated. The best value was used for further analysis.

The 5 m and 10 m sprints—Each participant performed three sprints of 10 m, with 1 min rest between trials [29]. Speeds were measured with two sets of three electronic photocells (Microgate^®^ Polifemo Radio Light, Bolzano, Italy), positioned 5 m (S5M) and 10 m (S10M) from the start line. Participants started from a stationary position, 0.5 m behind the start line [31]. Time was automatically activated as each participant passed the first gate at the 0 m mark and split times were recorded at 5 m (S5M) and 10 m (S10M). Trials where split times were not recorded were repeated. The best S5M and S10M were selected for further analysis.

**Figure 1:**
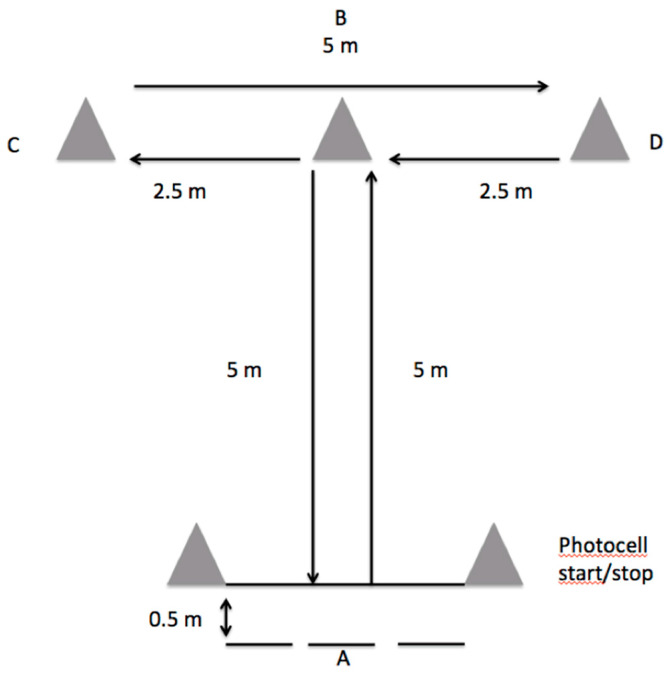
Modified agility test (MAT) test design.

Modified agility test—The modified agility test (MAT) was used to assess CODA ability [32]. Total distance of the test was 20 m. The participants’ movements during the MAT were as follows (Figure 1): (i) A-B movements (5 m): Participants sprinted forward to cone B and touched the top of it with the right hand; (ii) B-C movements (2.5 m): Moving laterally without crossing the feet, participants ran to cone C and touched its top with the left hand; (iii) C-D movements (5 m): Participants ran laterally to cone D and touched its top with the right hand; (iv) D-B movements (2.5 m): Participants moved back to cone B and touched its top with the left hand; (v) B-A movements (5 m): Participants ran backwards to line A. Trials where participants crossed their feet during B-C, C-D, and D-B movements, failed to touch the top of the cone, and/or failed to face forward throughout the tasks, were repeated. Each participant performed three trials interspersed with a 2 min rest period. One photocell gate was used to record the running time (Microgate^®^ Polifemo Radio Light, Bolzano, Italy). The best value was selected for further analysis.

Sit and reach (SAR) test—Each participant was asked to sit on the ground floor, with their bare feet placed vertically against a box. They had to lean forward with their arms and knees straight as far as possible and maintain that final position for 5 s [8,13,33]. Trials where the maintained position was less than 5 s or the knees were not fully extended, were repeated. Participants performed three trials interspersed with a 30 s rest period. The best value was considered for further analysis.

### 2.4. Statistical Analyses

The results are presented as means ± standard deviation (SD). All the variables presented a normal distribution according to the Kolmogorov–Smirnov test. Coefficients of variation (CV) of all the variables were calculated to determine the stability of the trials [CV = (SD/mean) × 100] [34]. One way ANOVA with the Bonferroni post hoc test was used to determine the differences among the different age groups. Effect size (ES) was calculated taking into account that the use of the *p* value alone provides no information about the size or direction of the effect or the range of feasible values [35]. The ES was calculated using the method proposed by Cohen [36]. Effects sizes lower than 0.2, between 0.2 and 0.49, between 0.5 and 0.79 and equal to or higher than 0.8 were considered trivial, small, moderate and high, respectively. A threshold value of 0.2 between-group standard deviation was set as the smallest worthwhile change and inference was then based on the disposition of the confidence interval for the mean difference to the smallest worthwhile effect; the probability (percent chances) that the true difference between groups is substantial or trivial was calculated using the magnitude-based inference approach. These percent chances were then qualified via probabilistic terms and assigned using the following scale: 25–74.9%, possibly; 75–94.9%, likely; 95–99.5%, very likely; ≤99.5%, most likely [35]. Pearson’s product-moment correlation coefficients (r) were used to determine the relationships between the different variables and 90% confidence limits (CL) were also calculated. The magnitude of correlation between tests were assessed with the following thresholds: <0.1, trivial; = 0.1–0.29, small; <0.3–0.49, moderate; <0.5–0.69, large; <0.7–0.89, very large; and <0.9–1.0, almost perfect [33]. The statistical analysis was carried out using the Statistical Package for Social Sciences (SPSS Inc., version 23.0, Chicago, IL, USA). The upper limit for statistical significance was set at *p* < 0.05.

## 3. Results

Handgrip isometric strength, vertical and horizontal jump performance, sprint, CODA and flexibility results of all the table tennis players are shown in Table 2. Except for the SAR test (CV = −22.71%), all the tests among repetitions CV were lower than 6.51%.

Table 3 shows the results obtained in the different tests for each group considering the table tennis players’ ages (i.e., U12, U14, U16, U20, Senior, and Older) and the differences among groups. Except for the senior and older groups, groups of a higher age presented better results than groups of a lower age such as shown by the U14 in comparison with U12 in all tests (ES = −0.70 to 1.98, moderate to large) except in SAR (ES = 0.19, trivial). The U16 group also obtained better results than U14 in all tests (ES = 0.77 to −2.31, moderate to large) except in SAR (ES = 0.19, trivial). The U20 group performed better than U16 in all the tests (ES = 0.73 to −1.53, moderate to large) except in the 5 m sprint test (ES = −0.02, trivial), 10 m sprint test (ES = −0.51, moderate) and SAR (ES = 0.11, trivial). Differences between the senior and U20 group were only found in CMJAS (ES = −0.82, large) and MAT (ES = 1.19, large), with the U20 group being better in both variables. The senior group performed better (ES = 0.94, large) in the MAT test than the older group.

Correlation coefficients were analyzed among the different tests (Table 4). SAR test did not show significant correlations with any test. Nevertheless, except between HANDG and S5M, in all tests (i.e., isometric strength, vertical and horizontal jump, S10M and CODA) significant most likely correlations were found (r = −0.53; ±0.14 CL to 0.87; ±0.05 CL, moderate to large).

## 4. Discussion

The purposes of this study were to analyze the table tennis players’ physical profile (i.e., handgrip isometric strength, vertical jump, horizontal jump, sprint, CODA, and flexibility) considering the age of the participants and to analyze the relationships among different physical capacities. Even though previous studies have analyzed the physical profile of table tennis players [16,17,28,37,38] the strength of this study lies in the assessment of a broader age range, because only a few of the aforementioned studies have considered a large age range. This novel approach allowed us to identify the differences on each of the capacities analyzed by this study in each of the age groups considered. The results of this study show that the U20 group performed significantly better than the other groups in every test, except for the SAR test. Moreover, moderate to high correlations were observed among the results obtained in the tests, except for the SAR test.

Table tennis is characterized by intermittent movement patterns that need a high level of strength, power, speed, and agility [9,10,39]. Regarding physical tests, the results of this study show a similar performance in the HANDG test compared to Spanish table tennis men players aged between 10–13 years and Korean table tennis players in the U20 category [8,28,40], but a higher performance than women participants between 20–25 years observed in previous studies in corresponding age groups (23% higher for the latter) [41]. On the contrary, the results of the present study show a lower performance (i.e., 12%) in the HANDG test than that observed in badminton players [42] and Turkish table tennis practitioners in the U12 category (i.e., 44%) [43]. In the same way, the results obtained in the CMJAS were also lower (i.e., 15%) than those previously observed in both women and men aged between 7 and 17 [17]. Nevertheless, Pradas de la Fuente et al. [37], Pullinger et al. [44], and Taş [43] got different results, showing different trends. Pradas de la Fuente et al. [37] observed a lower performance (i.e., 23%) in a previous study in the U12 category, but Pullinger et al. [44] and Taş [43] observed similar results in the U12 and U14 categories of table tennis players. As regards the HCMJAS, the table tennis players in the present study performed better than Korean table tennis players [40], had similar results comparing with U12 table tennis players [43] and U14 table tennis and tennis players [20,44], but worse than junior tennis players from the USA, in corresponding age groups [14]. The S5M and S10M results were similar to those observed in U14 table tennis [44] and tennis players, in corresponding age groups [20]. The results observed in the MAT tests were also better than those previously observed in university students, in corresponding age groups [32]. Nevertheless, participants in the present study performed worse than Korean table tennis players aged between 20 and 25 years [41]. The results observed in the SAR test were lower than previously observed in table tennis players, in corresponding age groups [8,40,43]. Differences in the results in the physical tests between studies might be due, in part, to the comparison of performance between different categories. Moreover, the different age ranges, when applied, in addition to the different moments of the season, the different loads and types of training in each study could have affected the results. For this reason, it could be interesting in future studies to analyze the physical condition of different racket sports players of similar age, competitive level, and moment of the season. Furthermore, future longitudinal studies are needed to observe the evolution of fitness status from young to older stages and determine whether the adherence to table tennis in childhood could lead to a healthier adulthood.

The results of the present study show that the results from the HANDG test increased according to age. These results coincide with previous studies where an increase in physical fitness was observed along with age, until a peak depending on the gender (i.e., age of 30 years for men and 50 years for women) [45,46]. Nevertheless, an increase in the vertical and horizontal jump, sprint capacity and CODA performance was observed until the age of 20. Thereafter, decreases in the previous parameters were evident [45,47]. Pradas de la Fuente et al. [17] showed similar results in vertical jump performance where significant differences were found in under 11 to junior (17 years old) table tennis players, the oldest ones being those who got better results comparing with the younger group. Previous studies have also found a direct correlation between jump performance and age, from 12 until 22 years of age [20,24,32] and from 12 to 18 years of age in the HANDG, CMJAS, and S10M tests [48]. Given the on-going deterioration from the age of 20 in vertical and horizontal jump, sprint capacity, and CODA, future studies should address whether specific strength, CODA, and acceleration training programs could delay the decrease in these abilities. Nevertheless, the maturity status of the players was not measured and these results were obtained without gender division. These aspects could have affected the obtained results in the different age groups. Future studies are therefore needed to consider the maturity status and the gender difference.

We observed a significant correlation (r = −0.53; ±0.14 CL to 0.87; ±0.05 CL, moderate to large, most likely) among the measured parameters (i.e., isometric strength, vertical and horizontal jump, sprint, and CODA), except for the SAR test. These results coincide with those obtained in previous studies where significant correlations between jump (horizontal and vertical) and sprint capacity in athletes from different sport disciplines such as handball, tennis, basketball or soccer [20,21,26,46,47,48,49,50,51,52] were observed. Besides, in a previous study [20] significant correlations between S5M and S10M and between CMJAS and sprint capacity (S5M and S10M) were reported in U14 tennis players. Similarly, previous studies [20,21,26,53,54] have reported significant correlations between CMJAS and HCMJAS in various sport disciplines (i.e., tennis, basketball, swimming), and other populations (i.e., physically active subjects, physical education students, healthy subjects) and age ranges (i.e., U12 and U20 years of age). As regards the HANDG, as we did, other researchers found significant correlations between this capacity with jump and sprint capacities [20,53]. Besides this, correlations between MAT with jump and sprint capacity have been reported by some researchers in different sport disciplines (i.e., soccer, tennis, swimming, basketball…) in some age groups (U12 to Senior) [27,32,50,55]. The results obtained in this study have shown the association between forearm isometric strength, vertical jump, horizontal jump, sprint capacity and CODA in table tennis players of different ages. The lack of a significant relationship between flexibility and other capacities suggests that flexibility is an independent capacity in this population as has been previously observed [25].

The present study is not without limitations. The main limitations of the study were that the maturation status of the participants was not measured, therefore it could affect the results of the study. Besides, no table tennis analyzing correlations among physical tests studies were found, making it difficult to compare the obtained results. Besides, the sample was also too small to give reference values in each age category. In the same vein, the obtained results have been shown without gender division, considering only the age of the participants. Despite, since women’s participation is low in each group, the results could have been affected due to gender differences. Additionally, the sample taken for women is significantly lower than in the case of men. Regarding correlations, the obtained results should be taken with caution because they may be influenced by the fact that some tests measure related capacities. Therefore, future studies should analyze the aforementioned variables with homogeneous groups divided by gender and with a larger sample, considering the maturation status.

The current results have several practical applications such as quantifying age-related changes in different physical capacities in table tennis players. This information could help in adapting training sessions, determining any health-related need in case any value is below average, or predicting future performance [13]. Concretely, in relation to performance, information about how players respond to particular tests assists coaches and physical trainers in setting benchmarks and developing effective training drills accordingly. Moreover, this information could be used for talent identification. On the other hand, in relation to health status, table tennis seems to be a useful tool to maintain adequate levels of different physical capacities. However, the particularities of this sport would require the use of adapted tests.

## 5. Conclusions

Forearm isometric strength improves with age. Nevertheless, jump, sprint, and CODA ability improve until 20 years of age and thereafter deteriorate (i.e., Senior and Older). Moreover, the relationship observed among forearm isometric strength, vertical jump, horizontal jump, sprint, and CODA ability suggests that these capacities are interrelated in table tennis players. Nevertheless, the lack of association between the sit and reach test with other capacities may indicate that flexibility is an independent capacity. These results show age-related changes in different physical tests, providing novel data about the fitness status of table tennis players, and they may open a window for future interventions using table tennis as a health promotion tool in different age groups. Furthermore, these findings could be useful for coaches in order to adapt training loads considering the players’ ages and biological changes occurring during maturation.

## Figures and Tables

**Table 1:** Table tennis players’ general characteristics depending on the age group and gender (mean ± SD).

	U12		U14		U16		U20		Senior		Older	
	M	F	M	F	M	F	M	F	M	F	M	F
Age (years)	10.00 ± 1.22	11.00 ± 0.00	12.50 ± 0.53	12.33 ± 0.58	14.50 ± 0.53	14.00 ± 0.00	17.79 ± 1.12	-	23.78 ± 2.11	23.33 ± 3.05	44.89 ± 9.44	30.00 ± 0.00
Body Mass (kg)	38.06 ± 5.36	42.50 ± 3.54	50.30 ± 11.42	44.33 ± 4.04	53.30 ± 9.06	47.00 ± 0.00	67.25 ± 10.97	-	77.44 ± 9.90	53.50 ± 6.06	81.56 ± 17.12	63.00 ± 0.00
BMI (kg·m^−2^)	19.10 ± 2.11	16.96 ± 3.02	18.87 ± 2.72	19.40 ± 2.33	19.48 ± 2.07	18.36 ± 0.00	22.30 ± 3.04	-	25.45 ± 3.44	20.26 ± 1.46	27.07 ± 5.09	21.78 ± 0.00
Body Height (m)	1.41 ± 0.08	1.59 ± 0.84	1.63 ± 0.07	1.51 ± 0.03	1.65 ± 0.10	1.60 ± 0.00	1.73 ± 0.07	-	1.75 ± 0.07	1.62 ± 0.05	1.73 ± 0.05	1.70 ± 0.00

SD = standard deviation; M = male; F = female; BMI = body Mass Index; U12 = under 12 years old; U14 = under 14 years old; U16 = under 16 years old; U20 = under 20 years old; Senior = Between 20 and 30 years old; Older = older than 30 years old.

**Table 2:** Handgrip isometric strength, vertical jump, horizontal jump, sprint, change of direction ability, and flexibility results of all the table tennis players.

	Min.	Max.	Mean ± SD	CV
Isometric strength				
HANDG (kg)	14.50	56.20	33.66 ± 11.74	6.51
Jump performance				
CMJAS (cm)	17.30	48.70	32.85 ± 7.73	5.56
HCMJAS (m)	1.30	2.40	1.92 ± 0.31	5.59
Sprint capacity				
S5M (s)	0.90	1.30	1.10 ± 0.09	3.48
S10M (s)	1.60	2.40	1.93 ± 0.16	2.68
Change of direction ability				
MAT (s)	5.40	9.20	6.72 ± 0.92	2.54
Flexibility				
SAR (cm)	−12.00	17.00	2.31 ± 7.91	−22.71

HANDG = handgrip isometric strength; CMJAS = vertical countermovement jump with arm swing; HCMJAS = horizontal countermovement jump with arm swing; S5M = 5 m sprint; S10M = 10 m sprint; MAT = modified agility test; SAR = sit and reach flexibility test; Min. = minimum value; Max. = maximum value; SD = standard deviation; CV = coefficient of variation.

**Table 3:** Handgrip isometric strength, vertical jump, horizontal jump, sprint, change of direction ability, and flexibility results by age group.

Variables	U12	U14 (ES U12-14)	U16 (ES U14-16)	U20 (ES U16-20)	Senior (ES U20-Senior)	Older (ES Senior-Older)
HANDG (kg)	17.82 ± 3.13	25.92 ± 4.07 (1.98)	34.95 ± 11.74 (0.77)	39.95 ± 6.38 (0.78)	42.25 ± 9.76 (0.23)	43.34 ± 6.68 (0.16)
CMJAS (cm)	22.84 ± 4.27	28.40 ± 6.57 (0.85)	36.32 ± 5.16 (1.54) (*p* = 0.02) *	39.55 ± 4.44 (0.73)	35.61 ± 4.78 (−0.82)	34.16 ± 7.76 (−0.19)
HCMJAS (m)	1.49 ± 0.22	1.72 ± 0.28 (0.82)	2.01 ± 0.22 (1.34) (*p* = 0.02) *	2.15 ± 0.10 (1.37)	2.09 ± 0.14 (−0.40)	2.01 ± 0.28 (−0.30)
S5M (s)	1.21 ± 0.07	1.13 ± 0.08 (−0.90)	1.04 ± 0.05 (−1.75)	1.04 ± 0.04 (−0.02)	1.05 ± 0.08 (0.15)	1.11 ± 0.10(0.56)
S10M (s)	2.11 ± 0.15	2.01 ± 0.14 (−0.70)	1.85 ± 0.07 (−2.31) (*p* = 0.04) *	1.81 ± 0.07 (−0.51)	1.83 ± 0.12 (0.19)	1.94 ± 0.16 (0.62)
MAT (s)	7.74 ± 0.77	7.11 ± 0.82 (−0.77)	6.31 ± 0.67 (−1.17)	5.86 ± 0.29 (−1.56)	6.26 ± 0.33 (1.19)	7.16 ± 0.96 (0.94)
SAR (cm)	2.00 ± 4.76	2.42 ± 8.72 (0.05)	3.9 ± 7.78 (0.19)	4.82 ± 8.36 (0.11)	1.55 ± 8.31 (−0.39)	−1.33 ± 9.50 (−0.30)

HANDG = handgrip isometric strength; CMJAS = vertical countermovement jump with arm swing; HCMJAS = horizontal countermovement jump with arm swing; S5M = 5 m sprint; S10M = 10 m sprint; MAT = modified agility test; SAR = flexibility sit and reach test; U12 = Under 12 years old; U14 = under 14 years old; U16 = under 16 years old; U20 = under 20 years old; Senior = between 20 and 30 years old; Older = older than 30 years old; ES = effect size. Significant differences (* *p* < 0.05) among groups.

**Table 4:** Correlation analysis (r; ±90% CL) among handgrip isometric strength, vertical jump, horizontal jump, sprint, change of direction ability, and flexibility tests for all the table tennis players.

	HANDG	CMJAS	HCMJAS	S5M	S10M	MAT	SAR
HANDG	-	0.64; ±0.12 ** 100/0/0 most likely	0.76; ±0.09 **	NS	−0.53; ±0.14 ** 0/0/100 most likely	−0.54; ±0.14 ** 0/0/100 most likely	−0.07 ± 0.2 8.0/51.8/40.2 possibly
CMJAS		-	0.83; ±0.06 ** 100/0/0 most likely	−0.71; ±0.10 ** 0/0/100 most likely	−0.74; ±0.09 ** 0/0/100 most likely	−0.63; ±0.12 ** 0/0/100 most likely	0.03 ± 0.22 8.1/57.8/14.1 possibly
HCMJAS			-	−0.71; ±0.10 ** 0/0/100 most likely	−0.71; ±0.10 ** 0/0/100 most likely	−0.79; ±0.08 ** 0/0/100 most likely	0.08 ± 0.2 43.4/49.8/6.8 possibly
S5M				-	0.87; ±0.05** 100/0/0 most likely	0.73; ±0.09 ** 100/0/0 most likely	-0.09±0.2 5.8/47.5/46.7 possibly
S10M					-	0.72; ±0.10 ** 100/0/0 most likely	−0.09 ± 0.2 5.8/47.5/46.7 possibly
MAT						-	−0.17 ± 0.19 1.2/26.6/72.2 possibly
SAR							-

HANDG = handgrip isometric strength; CMJAS = vertical countermovement jump with arm swing; HCMJAS = horizontal jump with arm swing; S5M = 5 m sprint; S10M = 10 m sprint; MAT = modified agility test; SAR = flexibility sit and reach test; CL = Confidence limits. Significant correlation (** *p* < 0.01) between variables.
